# Autobiographical

**Published:** 1893-01-07

**Authors:** 


The Hospital

An Institutional Journal of

Science, Medicine, Nursing, and Philanthropy.

*For Hospitals, Asylums, Infirmaries, Dispensaries, Medical Practitioners, Students, Nurses, and the Charitable Public.*

Vol. XIII.—No. 328.] [Jan 7, 1893.

### Autobiographical.

The year 1886 witnessed the birth of The Hospital. It was then a sheet of limited size, and consisted of only twenty-four pages, eighteen of which were literary matter, and six advertisements. For some time past, with a sheet greatly increased in size, the advertisements alone have filled more than twenty pages every week, and the literary matter amounted to forty pages on the 3rd ult., making that issue of The Hospital a paper of sixty pages instead of twenty-four.

Who reads the paper, and to whom does it appeal? On its first issue many persons were inclined to ask this question. Now everybody is much more disposed to ask why such a paper was not started half a century ago? The readers of the paper consist of all the persons who are minded to keep themselves thoroughly abreast of every department of hospital and medical affairs. Hospitals now take the most decided possible lead in everything that relates to the condition and progress of modern medicine. They have made modern medicine what it is. The Hospital is, therefore, in the first place, a journal of modern medicine—of modern medicine in its widest possible significance. It is a new type of medical journal, embodying and giving voice to an entirely new conception of medicine. There are many older medical journals of great excellence in every part of the world. We venture to say that they will all have, in no long time, to adopt the central principle of The Hospital. That principle is that everything that relates to man in his mind, body, or estate, is a part of medicine. Many of the older journals have taken the mere art of physic as the scope and limit of their purview. They have been technical journals in the narrowest sense; in the "journeyman's" sense, indeed. The Hospital, from the moment it decided that it must be a medical journal in the true sense of the word, decided that its motto must be, "The identity of man and medicine." The proper study of medicine is the universal and inclusive study of man. The purely technical and "journeyman's" view of the functions of medical journalism is no longer serviceable. The historical, philosophical, practical, and broadly human conception of medical activity can alone supply the journalism which will appeal to the cultured intelligence of the philosophic medical minds of the present and the near future.

The circulation of the paper is something of a phenomenon. More than half a million copies are published and sold annually. In saying this we are much within, as we wish to be, the actual figures which can be produced. Very early in the history of the journal it was seen that its success was to be marked and decided. A large circulation was rapidly gained. So long ago as December 1st, 1888, the *Pall Mall Gazette* bracketed The Hospital with the *British Medical Journal* and the *Lancet*, as being one of the three most influential and widely read of medical journals. Its weekly circulation was then 9,000, and it has steadily increased from that time. In April of the past year, notwithstanding the very extraordinary growth of the paper, so manifold were the interests which sought recognition and help in its pages that it was found necessary to add still further to its size and to increase its price. A large decrease of circulation was, of course, anticipated as the result of the doubling of the price. But the decrease, contrary to all expectation, was both small and temporary, and in a very short time the circulation began to rise again and has steadily continued to rise ever since, exactly as it did under the old penny *régime.*

The last great addition which has been made to the paper is the "Hospital Clinic." This is neither more nor less than an attempt to carry the hospital ward bodily into the consulting room of every private practitioner; or, to put it in another way, an attempt to furnish the private practitioner with the means of continuing his practical hospital studies to the very end of life. Every kind of hospital ward is, in its turn, laid under contribution; and every class of case, with its treatment from beginning to end, is presented to the practitioner's view. That this should be of immense service to the thoughtful mind goes without saying.

Previous to the addition of the Hospital Clinic we had found it necessary to add a "Medical Students' Department" to our columns, and still earlier a "Nursing Mirror Supplement." This supplement now extends to sixteen pages, and is almost as large as the whole paper was in its original form. Of those sixteen pages six are usually occupied by advertisements announcing nursing vacancies at hospitals in different parts of the United Kingdom and the world. When it is remembered that a few short years ago we were somewhat proud of six single advertisements of the kind which now fill six of our pages, it is manifest how widely THE HOSPITAL is read and accepted at hospitals, and how universally it is used as the great "institutional" medium of communication between them and hospital workers and officials of every class. Some people complain of what they call the "undue prominence given to mere nursing" in our pages. Will they give a little thought to this question—What is the most wide-reaching and practically serviceable of all the progressive advances made in general medicine during the last thirty years? There is but one answer: the development and perfection of the trained nursing department. The trained nurse is the doctor's right hand. We could almost better do without physic than without nursing. A large part of the practical medicine of modern times is, in one word, "nursing." True medical science, the science which deals with facts exactly as they are, sees these things, and seeing, gives them their due place and prominence in medical journalism.

THE HOSPITAL, we venture to think, has played a not inconsiderable, and certainly a not unworthy part in medical politics. Started on entirely independent grounds, and with an independent policy, it has, nevertheless, found that morality and the public interest demanded of it the unswerving support of legitimate medicine, and the equally unabated condemnation of unlicensed practice and every form of quackery. Briefly put, there is no necessity for unlicensed medicine, because every honest medical man finds himself at the fullest possible liberty to practise any form of medicine which he pleases, and which serves his patients, without let or hindrance from any of his professional brethren. What the profession objects to, and properly objects to, is the use of novel or professedly novel methods, not for the cure of patients, but for the puffing of practitioners. This is a mean kind of vulgarity and selfishness, which, much to its own honour, the medical profession steadily sets its face against. Some other callings would be none the worse if they took a leaf out of the medical book in this respect. Our sturdy loyalty to the legitimate interests of the profession has had its reward. Some of the most eminent members of the profession have contributed to our pages, including the present distinguished President of the Royal College of Physicians. Important medical associations have given us their confidence and sought our aid. There is a growing interest throughout the profession in the recognised originality and practicability of our methods and purposes. At the opening of the new Bristol Medical School in November last, Mr. Nelson Dobson, F.R.C S., Senior Surgeon to the hospital, in proposing the toast of "The Medical Press," eulogised the good work done by the *Lancet* and *British Medical Journal*, and now by THE HOSPITAL. All these things make us very grateful, and fill us with a satisfaction which few will be unkind enough to grudge.

No apology is made for this brief chapter of autobiography. There were reasons which seemed to us good and sufficient why it should be written. We venture to hope that the mass of our readers will be glad to be informed exactly how and where we stand. We have had no more than our just share of adversity, perhaps not so much. Our many friends have stood loyally by us, and we hope, should occasion require, our loyalty to them will not be either feeble or cold. We are more than grateful to all who have helped us, and we trust that neither now nor at any future time will they have reason to regret that they have contributed their quota to the building up and the permanent establishment of THE HOSPITAL.

